# Early access to medicinal products: potential and limits

**DOI:** 10.1186/1750-1172-9-S1-O24

**Published:** 2014-11-11

**Authors:** Pauline J Evers

**Affiliations:** 1 European genetic alliances Network (EGAN) and Dutch Federation of Cancer Patient Organisations (NFK), Utrecht, the Netherlands

## 

On average it takes 12 years and a billion euros to develop a new medicinal product from first discovery to availability for patients. Especially in the later stages of development, when promising phase 2 data are available it is difficult to accept for patients there is no general access. In order to make medicines available at an earlier stage and to optimise their development, new ways of taking medicines through the assessment procedures for registration and reimbursement are highly needed.

A new pilot, called adaptive [1,2] will start soon under responsibility of the European Medicines Agency. The idea behind is to give promising products marketing authorisation in a much earlier stage and collect more data, not only on safety but also on efficacy, after the product is on the market.

The biggest advantage is the early access for patients. In addition collection of data on efficacy on the daily use of the product (which might be different from the use in the preselected population in clinical studies) will give a better idea of the optimal use of the product in that daily setting. However, there are some downsides as well: what if a product after registration turns out not to be as good as we thought? Might this lead to stop of reimbursement or even withdrawal of the product and what would that mean for patients who are benefitting from the treatment? Secondly, we do already see now that reimbursement agencies and payers are not always convinced of the added value of newly registered products and decide not to reimburse. What would happen if products come to the market in a less mature stage? Would industry be prepared to launch their product at a lower price, since there is less robust evidence collected on their products? Would there be even more unequal access between European member states, reimbursement in some but not all?

The current system is no longer sustainable, so we need to start with these pilots. But we need to do it carefully, evaluating the pitfalls along the way, involving patients and physicians from the start as well as reimbursement agencies and payers. And we need to openly and honestly discuss the risks as mentioned above as well.
